# A Case of Non-cutaneous Kaposi Sarcoma

**DOI:** 10.7759/cureus.32394

**Published:** 2022-12-11

**Authors:** Noah Osei, Gerald Fletcher, Abosede Showunmi, Meena Ahluwalia

**Affiliations:** 1 Internal Medicine, Columbia University College of Physicians and Surgeons, Harlem Hospital Center, New York, USA; 2 Gastroenterology and Hepatology, Columbia University College of Physicians and Surgeons, Harlem Hospital Center, New York, USA; 3 Internal Medicine, Harlem Hospital Center, New York, USA; 4 Oncology, Columbia University College of Physicians and Surgeons, Harlem Hospital Center, New York, USA

**Keywords:** hiv aids, hoarseness, larynx, malnutrition, kaposi sarcoma

## Abstract

Kaposi sarcoma is a malignancy common in patients with acquired immune deficiency syndrome (AIDS). It is a proliferative soft-tissue tumor commonly manifesting as pigmented papules and nodules on the skin. Lesions can also appear on the mucosal lining of the oropharynx and other parts of the body such as the lymph nodes. Head and neck involvement in Kaposi sarcoma is not unusual; however, laryngeal involvement is not commonly seen. We report the case of a 31-year-old gentleman, a former smoker with AIDS, who developed a mass in the throat with progressive hoarseness of voice without stridor. An elective tracheostomy was done to protect his airway before performing a direct laryngoscopy with biopsy. Histopathology examination showed neoplastic spindle cells positive for CD31, erythroblast transformation specific-related gene, and human herpesvirus 8, consistent with Kaposi sarcoma. The diagnosis of laryngeal Kaposi sarcoma in immunodeficient patients requires a high index of suspicion, especially when it occurs without classical dermatological manifestation, an interesting feature in this report.

## Introduction

Kaposi sarcoma was first described in 1872 as a pigmented cutaneous sarcoma that presents most commonly on the lower extremities. Four epidemiologic-clinical subtypes of Kaposi sarcoma are African-endemic, epidemic acquired immune deficiency syndrome (AIDS)-related, classic, and iatrogenic Kaposi sarcoma. Although AIDS-related Kaposi sarcoma involves the head and neck, the primary laryngeal disease is uncommon [1]. A literature review showed only a handful of Kaposi sarcoma cases, approximately 77 to date, affecting the larynx [2].

Kaposi sarcoma is causally associated with infection of vascular endothelial cells by human herpesvirus 8 (HHV8) or Kaposi sarcoma herpesvirus (KSHV) infection, characterized by inhibition of apoptosis, angiogenesis, inflammation, and cellular proliferation. The interaction between the host immune dysfunction and the local inflammatory response to herpesvirus creates a conducive environment for the growth and progression of the disease [3]. KSHV infection alone is inadequate for the development of Kaposi sarcoma because it relies on immunosuppressive states such as those found in human immunodeficiency virus (HIV) infection [4], transplant patients, men having sex with men (MSM), and chronic systemic inflammatory conditions such as rheumatoid arthritis [5]. This is reflected in the higher incidence of Kaposi sarcoma in HIV-infected individuals compared to the general population [4]. Kaposi sarcoma frequently occurs at CD4 count <200 cells/µL, making it an AIDS-defining illness.

Diagnosing Kaposi sarcoma in the larynx can be challenging, especially without any cutaneous manifestations. Clinical presentation may include hoarseness, throat discomfort, urge to cough, aphonia, dysphagia, stridor, or complete airway obstruction. Radiographic evaluation with computed tomography (CT) scan of the neck helps to locate the laryngeal mass, as was done in this case. Biopsy of such vascular laryngeal mass is required for a definitive diagnosis but is associated with risks and severe bleeding [6].

Highly active antiretroviral therapy (HAART) is an indispensable component of the treatment for AIDS-associated Kaposi sarcoma and has resulted in a substantial decline in the incidence of Kaposi sarcoma [7]. Close clinical supervision is warranted due to the risk of Kaposi sarcoma inflammatory cytokine syndrome (KICS), a potentially fatal complication resulting from systemic immune reconstitution inflammatory response during HAART initiation or resumption [8]. Other treatment modalities include cryotherapy, surgery, radiotherapy, chemotherapy, and immunotherapy. Due to the increased risk associated with laryngeal Kaposi sarcoma lesions, emergent interventions such as tracheostomy are necessary to prevent acute or impending airway obstruction. However, tracheostomy insertion may increase mortality risk due to fatal bleeding [9].

## Case presentation

A 31-year-old African American man, newly diagnosed with HIV, presented to the hospital with a five-month history of throat mass associated with hoarseness of voice without stridor or dysphagia. He also reported a 30-pound weight loss over a six-month period, lethargy, and anorexia. He had never sought medical attention in the past. Physical examination showed temporal wasting, a muffled voice, and a mass protruding from the posterior pharynx. Submandibular lymphadenopathy was palpable. No skin lesions were found on examination.

**Figure 1:**
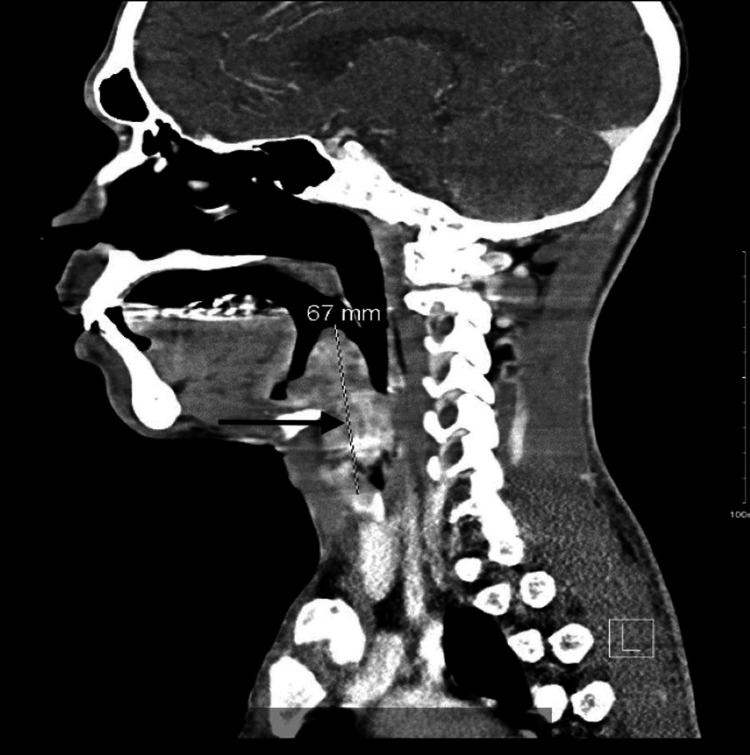
Contrast-enhanced computed tomography images (sagittal view) showing a lobulated mass arising from the epiglottis and extending to the oro- and hypopharynx. The mass lesion causes near-complete obstruction of the upper airway.

**Figure 2:**
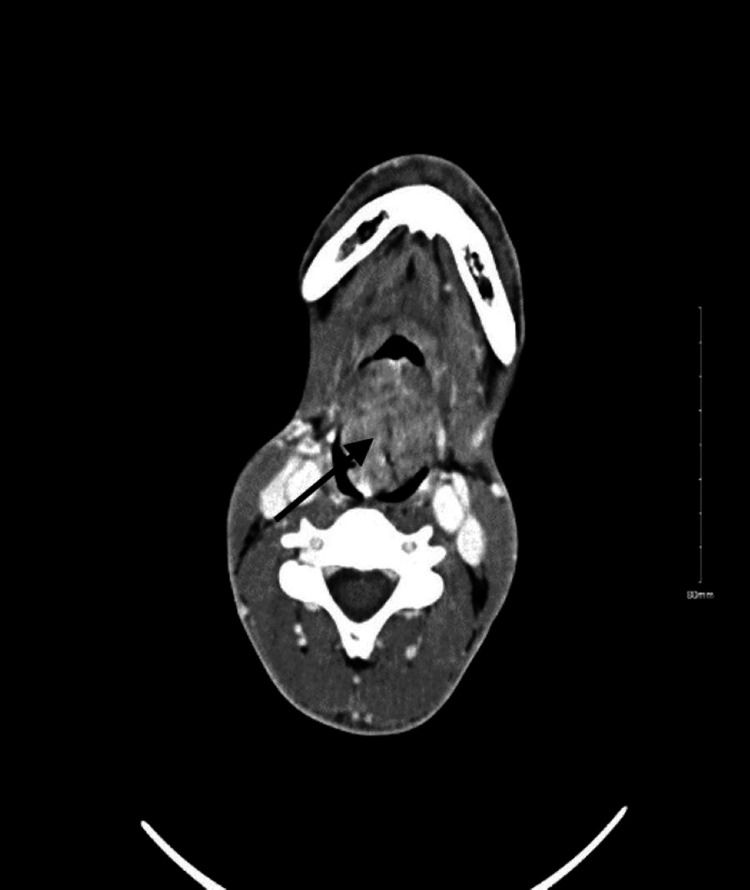
Contrast-enhanced computed tomography images (transverse view) showing a lobulated mass arising from the epiglottis and extending to the oro- and hypopharynx. The mass lesion causes near-complete obstruction of the upper airway.

**Figure 3:**
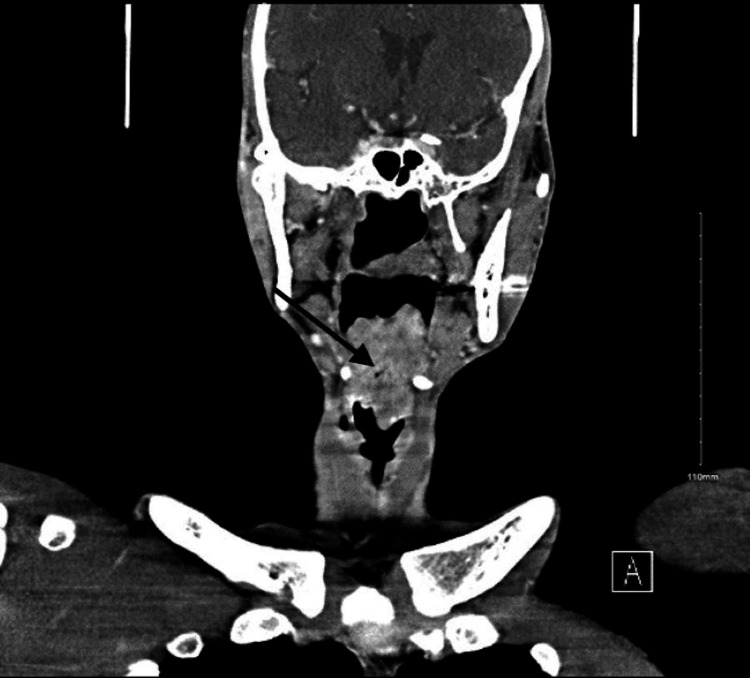
Contrast-enhanced computed tomography images (coronal view) showing a lobulated mass arising from the epiglottis and extending to the oro- and hypopharynx. The mass lesion causes near-complete obstruction of the upper airway.

Routine blood tests showed a CD4 count of 28 cells/µL and an HIV viral load of 221,000 copies/mL. CT scan of the neck showed a large lobulated, heterogeneous, enhancing, mass-like lesion, likely arising from the epiglottis measuring approximately 4.2 × 3.7 × 6.7 cm, extending to the hypopharynx and supraglottic/glottic larynx, and effacing the bilateral vallecula and left pyriform sinus. The lesion was causing near-complete obstruction of the upper airway at the level of the supraglottic larynx, posterior to the epiglottis (Figures 1-3).

**Figure 4:**
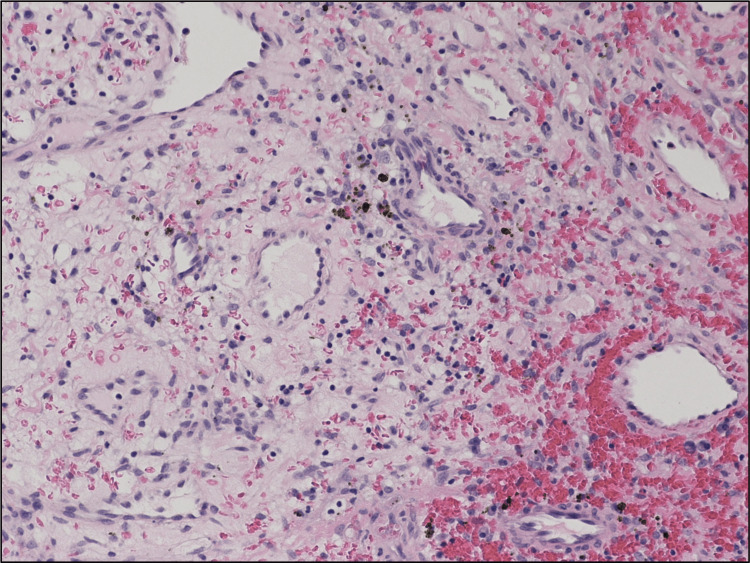
Neoplastic spindle cells present, along with extravasation of erythrocytes (hematoxylin and eosin stain).

**Figure 5:**
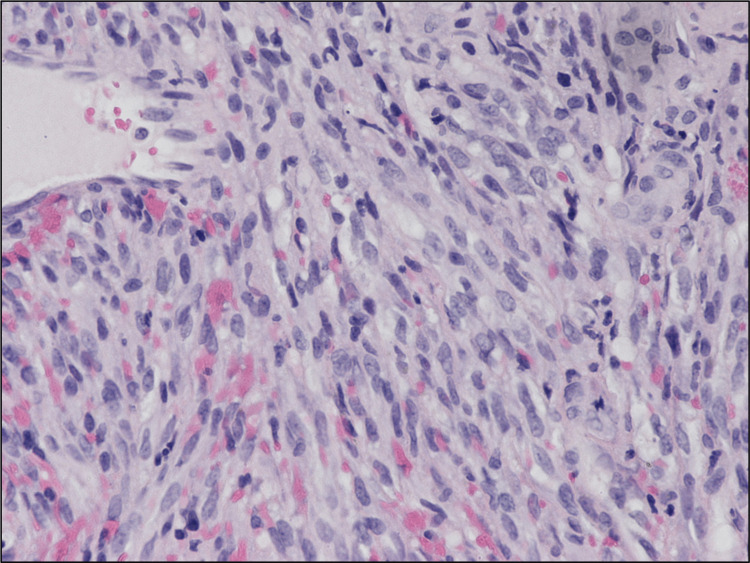
Intracellular and extracellular hyaline globules (hematoxylin and eosin stain).

A multidisciplinary team meeting recommended elective tracheostomy before laryngoscopy due to the high risk of airway compromise. The tracheostomy tube was secured before direct laryngoscopy, which revealed a large 5 cm exophytic, friable, necrotic mass centered in the midline vallecula. Sections of the laryngeal mass showed a monotonous proliferation of neoplastic spindle cells with a somewhat fascicular growth pattern, frequent ectatic vascular spaces, and extravasation of red blood cells (Figures 4, 5). Occasional intracellular and extracellular hyaline globules were present, along with scattered lymphocytes (Figure 5).

**Figure 6:**
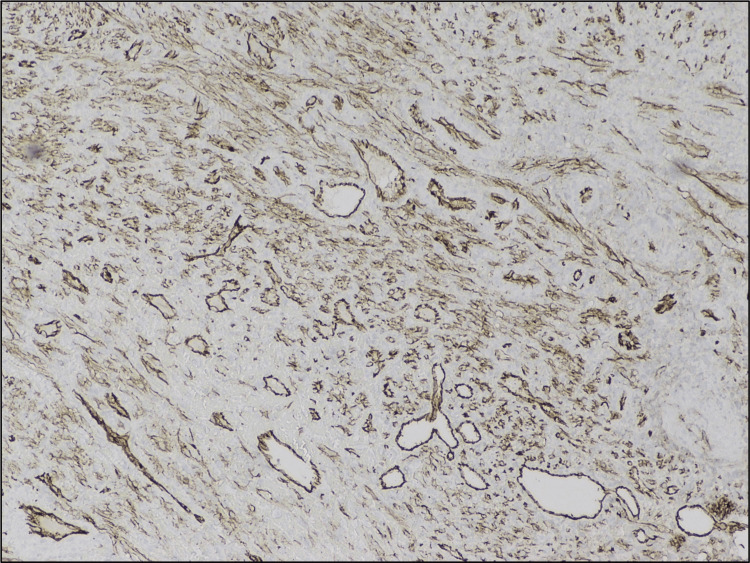
CD31 positive.

**Figure 7:**
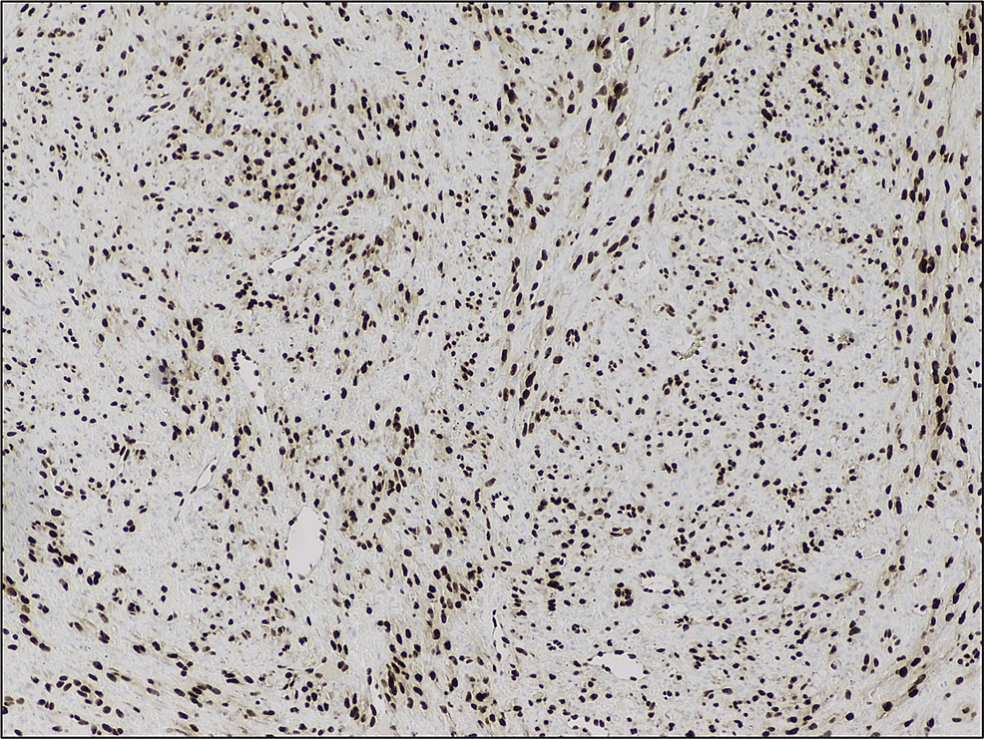
Erythroblast transformation specific-related gene positive.

**Figure 8:**
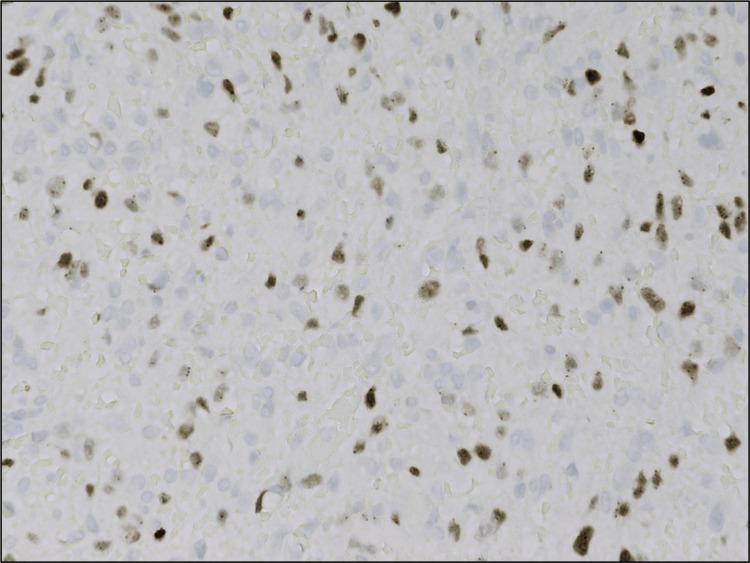
Human herpesvirus 8 positive.

The neoplastic spindle cells were positive for CD31 (Figure 6), erythroblast transformation specific-related gene (ERG) (Figure 7), and LANA stain for HHV8 was positive (Figure 8). There was no immunomorphologic evidence of lymphoma. These findings were consistent with Kaposi sarcoma.

He was treated with bictegravir, emtricitabine, and tenofovir alafenamide as antiretroviral therapy. In addition, he was started on oral fluconazole and Bactrim as prophylaxis for AIDS-associated opportunistic infections. He underwent a percutaneous endoscopic gastrostomy (PEG) tube placement due to the dysphagia resulting from the mass effect of the lesion on the esophagus. The patient was later referred to an oncology center where he is currently receiving systemic chemotherapy consisting of liposomal anthracycline (doxorubicin) in addition to HAART. His CD4 count improved from 28 cells/µL at the time of admission to 115 cells/µL at the time of transfer.

## Discussion

Visceral involvement of AIDS-related Kaposi sarcoma is uncommon, and laryngeal involvement without cutaneous lesions rarely occurs. To our knowledge, a recent systematic review found only 77 published case reports of Kaposi sarcoma of the larynx, predominantly in males and mostly arising in the supraglottic region [2]. The presenting symptoms can range from hoarseness of voice, which was the presenting symptom in this patient, and dysphagia to stridor or complete airway obstruction [10]. Pain, bleeding, and speech abnormalities are possible symptoms. Examination with laryngoscopy can assist with the diagnosis because it allows biopsies to be taken and analyzed.

Given the highly vascularized nature of the tumor, a biopsy of suspected laryngeal Kaposi sarcoma is usually associated with a high risk of airway compromise from significant bleeding and edema [6]. In this case, the airway was secured by inserting a tracheostomy tube before direct laryngoscopy and biopsy. The patient was monitored closely for bleeding, and no complications occurred. Histologically, Kaposi sarcoma is a spindle-shaped cell with vascular channels filled with red blood cells. The staining for endothelial markers CD31, CD34, ERG, and D2-40 confirms Kaposi as a vascular tumor, as was demonstrated in this patient [11]. Monoclonal antibody to HHV8 LNA-1 is a cost-effective and reliable method to detect HHV8 in fixed human tissues by immunohistochemistry. It enables the differentiation of Kaposi sarcoma from other histologically similar entities [12].

The AIDS Clinical Trials Group (ACTG) guidelines allow for better staging of AIDS-associated Kaposi sarcoma based on the extent of the disease (T), the severity of immunodeficiency (I), and the presence of systemic symptoms (S). Based on these criteria, patients are classified as good or poor risk [13]. Treating the underlying HIV infection can effectively induce tumor regression in most patients considered a good risk.

The widespread use of HAART as an essential component of the treatment regimen for AIDS-associated Kaposi sarcoma has yielded excellent results. It helps in shrinking existing cancer lesions and prevents dissemination. Since its introduction, HAART has significantly reduced disease incidence and improved the survival rate of individuals with AIDS-associated Kaposi sarcoma [7]. Our patient received a combination of bictegravir, emtricitabine, and tenofovir alafenamide as his antiretroviral treatment, improving his CD4 count from 28 cells/µL to 115 cells/µL six weeks after initiation. His viral load also decreased from 221,000 copies/mL to 265 copies/mL before his transfer to another facility for definitive treatment. Close clinical monitoring is required during the start of HAART usage due to the risk of Kaposi sarcoma flare resulting from immune reconstitution inflammatory syndrome [8].

Individuals classified as poor risk according to the ACTG criteria, an example being this index case, generally benefit from a combination of antiretroviral drugs and an additional therapy such as chemotherapy [13]. Drugs such as bleomycin, vinblastine, vincristine, hydroxydaunomycin, adriamycin, and etoposide have previously been used against Kaposi sarcoma. Currently, liposomal anthracyclines (pegylated liposomal doxorubicin and liposomal daunorubicin) and taxanes (paclitaxel) constitute the backbone of systemic cytotoxic therapy against Kaposi sarcoma [3], which is what our patient is receiving at the time of this report. Newer treatments such as interleukin 12, interferon alpha, and tyrosine kinase inhibitors such as imatinib have shown varying degrees of response in treating Kaposi sarcoma. Rapamycin (sirolimus), a PI3K/Akt/mTor inhibitor pathway, has been effective in treating post-transplant and classic Kaposi sarcoma and is currently being investigated for HIV-related Kaposi sarcoma [3].

Studies have shown that patients undergoing chemotherapy or radiotherapy with head and neck cancers are at an increased risk of significant weight loss and malnutrition due to impaired swallowing and concomitant odynophagia from mucosal injury during treatment. They lead to frequent hospitalizations, poor compliance with treatment, and reduced quality of life. The early use of PEG tubes for enteral feeding reduces the impact of systemic therapy on weight loss and nutritional status [14,15]. Considering this, our team placed a PEG tube to assist the patient in meeting the needed calories and nutritional requirements essential for patient strength and promoting good host response to therapy.

## Conclusions

Kaposi sarcoma, a highly vascular tumor, is commonly seen in patients with AIDS as cutaneous lesions. Laryngeal involvement is uncommon but often associated with AIDS or a history of immune suppression. Diagnosis requires a high index of suspicion in the absence of cutaneous lesions. Biopsy must be cautiously performed due to the risk of bleeding and airway obstruction. HAART is fundamental in the treatment in addition to systemic therapy and good nutritional support.

## References

[1] Naimi Z, Mahjoubi K, Adouni O, Abidi R, Driss M, Nasr C (2020). Kaposi's sarcoma of the larynx: an unusual location in an HIV-negative patient (a case report). Pan Afr Med J.

[2] Barron K, Omiunu A, Celidonio J (2022). Kaposi sarcoma of the larynx: a systematic review. Otolaryngol Head Neck Surg.

[3] Douglas JL, Gustin JK, Moses AV, Dezube BJ, Pantanowitz L (2010). Kaposi sarcoma pathogenesis: a triad of viral infection, oncogenesis and chronic inflammation. Transl Biomed.

[4] Mesri EA, Cesarman E, Boshoff C (2010). Kaposi's sarcoma and its associated herpesvirus. Nat Rev Cancer.

[5] Seleit I, Attia A, Maraee A, Samaka R, Bakry O, Eid E (2011). Isolated Kaposi sarcoma in two HIV negative patients. J Dermatol Case Rep.

[6] Mochloulis G, Irving RM, Grant HR, Miller RF (1996). Laryngeal Kaposi's sarcoma in patients with AIDS. J Laryngol Otol.

[7] Grabar S, Abraham B, Mahamat A, Del Giudice P, Rosenthal E, Costagliola D (2006). Differential impact of combination antiretroviral therapy in preventing Kaposi's sarcoma with and without visceral involvement. J Clin Oncol.

[8] Dumic I, Radovanovic M, Igandan O (2020). A fatal case of Kaposi sarcoma immune reconstitution syndrome (KS-IRIS) complicated by Kaposi sarcoma inflammatory cytokine syndrome (KICS) or multicentric Castleman disease (MCD): a case report and review. Am J Case Rep.

[9] Beitler AJ, Ptaszynski K, Karpel JP (1996). Upper airway obstruction in a woman with AIDS-related laryngeal Kaposi's sarcoma. Chest.

[10] Mohd Tahir J, Marina MB, Gopalan KN, Primuharsa Putra SH (2010). A rare case of laryngeal Kaposi's sarcoma. Bangladesh J Med Sci.

[11] Rosado FG, Itani DM, Coffin CM, Cates JM (2012). Utility of immunohistochemical staining with FLI1, D2-40, CD31, and CD34 in the diagnosis of acquired immunodeficiency syndrome-related and non-acquired immunodeficiency syndrome-related Kaposi sarcoma. Arch Pathol Lab Med.

[12] Patel RM, Goldblum JR, Hsi ED (2004). Immunohistochemical detection of human herpes virus-8 latent nuclear antigen-1 is useful in the diagnosis of Kaposi sarcoma. Mod Pathol.

[13] Krown SE, Metroka C, Wernz JC (1989). Kaposi's sarcoma in the acquired immune deficiency syndrome: a proposal for uniform evaluation, response, and staging criteria. AIDS Clinical Trials Group Oncology Committee. J Clin Oncol.

[14] Madhoun MF, Blankenship MM, Blankenship DM, Krempl GA, Tierney WM (2011). Prophylactic PEG placement in head and neck cancer: how many feeding tubes are unused (and unnecessary)?. World J Gastroenterol.

[15] Anwander T, Bergé S, Appel T (2004). Percutaneous endoscopic gastrostomy for long-term feeding of patients with oropharyngeal tumors. Nutr Cancer.

